# A Rare Case of Tolosa-Hunt-Like Syndrome in a Poorly Controlled Diabetes Mellitus

**DOI:** 10.1155/2016/9763621

**Published:** 2016-03-20

**Authors:** Glenmore Lasam, Sakshi Kapur

**Affiliations:** ^1^Department of Medicine, Overlook Medical Center, Summit, NJ 07901, USA; ^2^Section of Hospital Medicine, Overlook Medical Center, Summit, NJ 07901, USA

## Abstract

We report a case of a 50-year-old female with diabetes mellitus who presented with progressive second, third, fifth, sixth, and eighth cranial nerve palsy. Diagnostic investigation revealed hyperglycemic state, and brain imaging showed a right cavernous sinus enhancement suggestive of and consistent with Tolosa-Hunt syndrome. The patient was started on steroids with tight glycemic control for eight weeks; subsequently, the cranial nerve palsies resolved as well as documented resolution of the right cavernous sinus enhancement.

## 1. Introduction

Tolosa-Hunt-like syndrome in diabetes mellitus is a challenging phenomenon that may pose a diagnostic dilemma and, subsequently, a treatment modality.

## 2. Case Presentation

A 50-year-old woman with an uncontrolled type II diabetes mellitus presented with right- sided eyelid droop, right facial weakness and sag, right-sided forehead paresthesia, and difficulty abducting the right eye for at least seven days. She also had chronic right eye blurry vision and a decreased right ear hearing acuity. She denied any weakness in her arms and legs, slurred speech, or difficulty swallowing. There is no history of sudden fall, trauma, camping, hiking, recent illness, sick contact, or recent travel. She also had history of hypertension, and it has been controlled. She has not been compliant with her diabetic regimen and, currently, was not taking glycemic medications upon presentation. She was nonsmoker, nonalcoholic, and nonillicit drug user. Her examination was remarkable for optic, oculomotor, trigeminal, facial, and vestibulocochlear dysfunction. Pupils were equally reactive to light and accommodation with no note of anisocoria. There was no appreciable diabetic nor hypertensive retinopathy changes on fundoscopy. Extraocular muscles were full and intact except for inability to fully abduct the right eye. There was significant right ptosis, right-sided facial droop, and decreased sensation to pain, touch, and temperature in the distribution of the ophthalmic division of the right trigeminal nerve. She has diminished hearing acuity on the right ear. Motor strength was normal symmetrically on her extremities. Vibration, proprioception, and sensation to other body parts have been intact. Deep tendon reflexes were normal. She was able to ambulate with steady gait with no note of ataxia or other cerebellar dysfunction. During her course, she also complained of pressure-like intermittent right-sided headache associated with steady periorbital pain with retroorbital extension.

**Figure 1:**
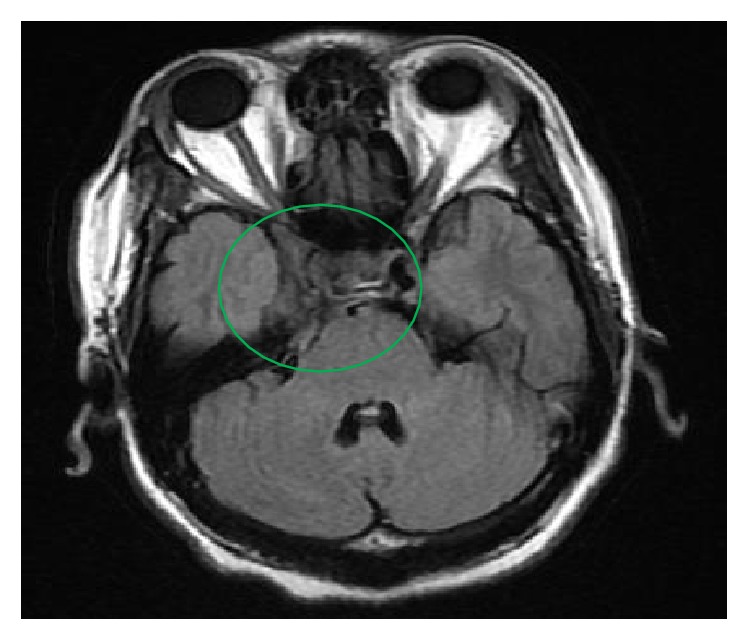
Initial cranial MRI revealing abnormal enhancement involving the right cavernous sinus area extending along the tracks of the right fifth, seventh, and eighth cranial nerves.

**Figure 2:**
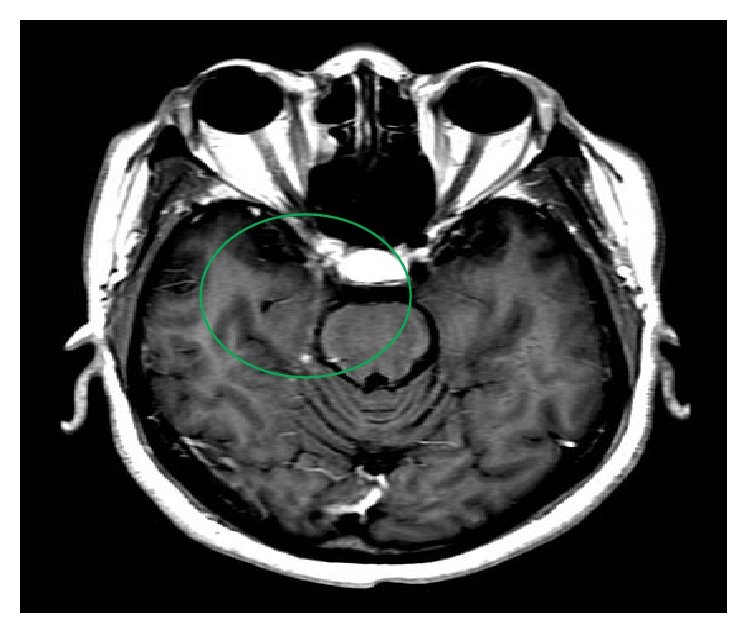
Subsequent cranial MRI showed resolution of the abnormal enhancement in the cavernous sinus area seen on the initial cranial MRI.

Her glycosylated hemoglobin was 12.4%, and her initial blood sugar readings range from 200 to 300 mg/dL which improved to 100–200 mg/dL with initiation of insulin regimen. Investigatory laboratory studies were normal including complete blood count, comprehensive metabolic panel, lipid profile, thyroid function studies, coagulation studies, serum cyanocobalamin levels, serum rapid plasma reagin, human immunodeficiency virus antibody, serum angiotensin converting enzyme, toxoplasmosis antibody titer, cryptococcal antigen titer, cytomegalovirus deoxyribonucleic acid polymerase chain reaction, and anti-neutrophil cytoplasmic antibodies. Erythrocyte sedimentation rate was 38 mm/hr, while C-reactive protein was normal. She had past exposure to Epstein-Barr virus and parvovirus, as evidenced by reactive IgG antibody titers. Cerebrospinal fluid studies were unremarkable including cell count, culture, angiotensin converting enzyme levels, acid-fast bacilli culture, Epstein-Barr virus polymerase chain reaction, lyme polymerase chain reaction, venereal disease research laboratory test, oligoclonal bands, myelin basic protein, and flow cytometry for lymphoma. Mantoux test was negative. Her chest, abdomen, and pelvis computed tomography were unremarkable. Brain magnetic resonance imaging showed abnormal enhancement involving the right cavernous sinus extending along the tracks of the right fifth, seventh, and eighth cranial nerves (Figure 1). Also, it revealed severe narrowing and encasement of the right cavernous portions of the internal carotid artery, confirmed by angiography, which disclosed right carotid artery displacement likely from the mass effect of the lesion. A right orbital proptosis was seen on computed tomography of the sinus. Tolosa-Hunt-like syndrome has been considered because of the patient's clinical manifestations, nonrevealing laboratory test, unremarkable cerebrospinal fluid analysis, and imaging findings highly suggestive of inflammatory process, likely consistent with the condition. Malignancy, neurosarcoid, and autoimmune diseases have been ruled out. She was started on prednisone 60 mg daily with note of considerable relief of retroorbital pain and headache with noticeable improvement in the right ptosis within two days of therapy. Prednisone was continued on discharge and was tapered slowly during her eight-week course of therapy. She was also maintained on tight glycemic control with insulin. On her follow-up after eight weeks of treatment, she showed complete resolution of ophthalmoplegia, ptosis, headache, and right-sided forehead paresthesia as well as significant improvement in her hearing and visual acuity. Repeat cranial magnetic resonance imaging was normal (Figure 2). She has no recurrence of the above complaints on subsequent health maintenance evaluations.

## 3. Discussion

Tolosa-Hunt syndrome (THS) is a rare condition that is hallmarked by painful ophthalmoplegia described as recurrent unilateral orbital pain and ipsilateral third, fourth, and/or sixth cranial palsies which significantly improves with steroids. Tolosa in 1954 elucidated the syndrome's clinical features [1] while Hunt et al. in 1961 stressed the effectiveness of corticosteroid treatment [2]. The annual incidence of the syndrome has been reported to be one case per million per year [3]. The etiology of THS has been attributed to nonspecific inflammatory process of uncertain cause that has been confined to the septa and wall of the cavernous sinus described as infiltration of lymphocyte and plasma cell, formation of giant cell granulomas, and fibroblast proliferation [1, 2].

The onset of the disease is the continuous pain induced by pressure and dysfunction of the structures within the cavernous sinus, superior orbital fissure, or the apex of the orbit. Ophthalmoparesis ensues when granulomatous inflammation in the cavernous sinus extends to oculomotor, trochlear, and abducens cranial nerves, whereas paresthesia of the forehead occurs with involvement of the superior division of the trigeminal nerve. Seventy-five percent of patients who manifest painful ophthalmoplegia will not have the diagnosis of THS [4, 5]. Painful ophthalmoplegia is the consequence of the mass effect on the cavernous sinus which includes trauma, vascular malformation, neoplasm, infection, and inflammation including Tolosa-Hunt syndrome, Wegener's granulomatosis, or orbital pseudotumor [6]. Aside from anatomical compressive lesions, painful ophthalmoplegia can also be effected by giant cell arteritis, ophthalmoplegic migraine, or a diabetic ophthalmoplegia [7]. Vascular conditions and tumors are the predominant etiologies of painful ophthalmoplegia.

The concomitant occurrence of Tolosa-Hunt syndrome and diabetes mellitus especially if poorly controlled has not been clearly elucidated and reported. Cranial neuropathies in diabetic patients are extremely rare and occur in older patients with poorly controlled diabetes [8]. Hyperglycemia and the eventual ischemic occurrence stemming from diabetic macroangiopathic changes have been postulated as the most common causes of the cranial nerve palsies especially the oculomotor nerve [9].

The syndrome has been characterized by episodic orbital pain accompanied by paralysis of the oculomotor, trochlear, and/or abducens cranial nerves that tends to resolve spontaneously but may remit and relapse [10]. The pain is typically unilateral and periorbital in location but can extend in the retroorbital, temporal, and frontal areas and has been characteristically described as a steady, intense, lancinating, gnawing, stabbing, or boring pain. The oculomotor nerve has been involved frequently in 85% of cases, abducens nerve in 70% of cases, trochlear nerve in 29% of cases, and ophthalmic division of trigeminal nerve in 30% of cases [11], and periarterial sympathetic fibers were in 20% of cases that causes Horner's syndrome [12, 13]. It has the same predilection in terms of frequency to both men and women and can affect any age group. Optic nerve disturbance is attributed to lesion extension to the orbital apex which contributes to optic disc swelling or pallor [6], thickened optic nerve due to edema [14], and orbital venous congestion [15] that may cause minimal visual decline though variable. In addition to ophthalmoplegia, though infrequent, inflammation extending beyond the cavernous sinus would affect the maxillary [16] and mandibular branch of the trigeminal nerve [17] and the facial nerve [18].

Specific diagnostic criteria have been recommended by the International Headache Society which includes unilateral headache; granulomatous inflammation of the cavernous sinus and superior orbital fissure or orbit seen on magnetic resonance imaging or biopsy; paresis of one or more of the ipsilateral third, fourth, and/or sixth cranial nerves; evidence of causation demonstrated by both headache preceded by oculomotor paresis by less than 2 weeks or developed with it and headache that is localized around the ipsilateral brow and eye; and symptoms not better accounted for by an alternative diagnosis [9]. The diagnosis of the syndrome is based upon the clinical manifestation in association with neuroimaging, lumbar puncture, array of laboratory test, and swift response to glucocorticoids. Due to the difficulty accessing the cavernous sinus to obtain a tissue sample with potential harm to the patient, direct biopsy is rarely advocated. Both typical symptoms and imaging findings predicted a diagnosis of THS with high sensitivity of 95.8% and 100%, respectively, but low specificity of 47.2% and 28.6%, respectively [19]. Magnetic resonance imaging usually reveals an enhancing soft tissue lesion within the cavernous sinus, an increase in size and lateral bulging of the anterior cavernous sinus contour, an extension of the lesion to the superior orbital fissure and orbital apex, and a note of internal carotid artery narrowing [20]. Cerebrospinal fluid studies (glucose, protein, cell count with differential, culture, and cytology) including lyme and syphilis serology, angiotensin converting enzyme, and mycobacteria culture are normal.

Corticosteroid has been utilized for diagnostic purposes as well as therapeutic modality and the rapid response to pain relief within a day and up to three days assists in committing to the diagnosis [7, 21, 22]. It is started initially as high dose for a period of two to four weeks and then tapered gradually over at least four to six weeks [4, 23]. Over the succeeding two to eight weeks, resolution of ophthalmoplegia and reversion of imaging abnormalities also help in leaning towards the diagnosis [11, 24], though other diseases like vasculitis and lymphoma also respond to steroids as evidenced by improvement of symptoms and imaging findings [25]. The clinical manifestations of the syndrome may resolve spontaneously even if left untreated after about eight weeks [2, 6]. Though steroids accelerated orbital pain resolution, it has not been proven that ophthalmoplegia improves rapidly with or without treatment [22]. Because of the Tolosa-Hunt syndrome's classic quick response to steroids, limited attention has been made to explore other possible treatment options like tight glycemic control in patients with poorly controlled diabetes mellitus which in a way may have contributed to the resolution of the syndrome's manifestations. Continued optimal glycemic control even during steroid taper normalized the cranial nerve palsy by four weeks with complete resolution of orbital apex lesion after twelve weeks confirmed by magnetic resonance imaging [26]. The clinical presentation of our patient especially the involvement of optic, facial, and vestibulocochlear cranial nerves on top of the classic oculomotor, trochlear, trigeminal, and abducens cranial nerve palsies leaned towards a Tolosa-Hunt-like syndrome. Furthermore, the treatment instituted to our patient was identical to the standard treatment for classic THS which inclined towards the possibility of an evolving variant of the syndrome.

## 4. Conclusion

Tolosa-Hunt-like syndrome in DM necessitates early initiation of corticosteroids with tight glycemic control to resolve symptoms and prevent neurological sequela especially cranial nerve palsies.
